# Outcomes Following Percutaneous Coronary Intervention in Saphenous Vein Grafts With and Without Embolic Protection Devices: A Systematic Review and Meta-Analysis

**DOI:** 10.3389/fcvm.2021.726579

**Published:** 2022-01-21

**Authors:** Jianhong Yu, Jianhai Zhang, Jianchao Ni, Weiqing Shou, Yuanyuan Fang, Suna Fu

**Affiliations:** Department of Geriatrics, Affiliated Hospital of Shaoxing University, Shaoxing, China

**Keywords:** embolic protection devices, meta-analysis, percutaneous coronary intervention, saphenous vein graft, systematic review

## Abstract

**Objective:**

This study aimed to review studies comparing outcomes following percutaneous coronary intervention (PCI) in saphenous vein grafts (SVG) with and without embolic protection devices (EPD).

**Methods:**

Databases including PubMed Central, Cochrane Library, EMBASE, CINAHL, MEDLINE, Google Scholar, ScienceDirect, and Scopus were searched from January 1964 to April 2021. We used the Cochrane risk of bias tool and the Newcastle Ottawa scale to assess the quality of published studies based on study design. From the results, we carried out a meta-analysis with a random-effects model and reported pooled odds ratio (OR) with 95% CI.

**Results:**

In total, 11 studies were analyzed that included 79,009 total participants. EPD use had significantly lower odds of mortality (pooled OR = 0.69; 95% CI: 0.5–0.94). There was no significant difference in terms of major adverse cardiovascular events (MACE) (pooled OR = 0.83; 95% CI: 0.67–1.03), target vessel revascularization (pooled OR = 1; 95% CI: 0.95–1.05), periprocedural (pooled OR = 1.12; 95% CI: 0.65–1.9) and late myocardial infarction (MI) (pooled OR = 0.79; 95% CI: 0.55–1.14) with or without EPD for PCI in SVG patients.

**Conclusion:**

Although not statistically beneficial for MACE, target vessel revascularization, periprocedural, and late MI, EPD use does appear to significantly reduce mortality for the patients undergoing PCI in SVG. Clinicians might consider using EPD for such patients to reduce the burden of post-procedural morbidity and mortality.

## Introduction

The percutaneous coronary intervention of the saphenous vein grafts has been associated with several periprocedural complications compared to the native vessel percutaneous coronary intervention (PCI). This is attributed to the atheroma distal embolization from the degenerative graft lesion leading to a no-reflow phenomenon and periprocedural myocardial infarction (MI) (1, 2). The latest guidelines recommend using embolic protection devices (EPD) as a Class I indication (with the level of evidence B) for the saphenous vein grafts (SVG) intervention whenever possible and technically feasible for minimizing the distal embolization (3). However, this recommendation is based on a single randomized controlled trial (RCT), which compared distal EPD against no EPD in SVG intervention. Several recent publications on EPD use in SVG intervention have been found to show conflicting results (4–7). Data utilized from a large-scale National Cardiovascular Data Registry (NCDR—CathPCI Registry) showed no additional benefit with the routine use of EPD during the SVG intervention (8). The study has reported a significant association between the use of EPD and a higher incidence of periprocedural complications (8).

Despite modifications and the development of upgraded versions, the use of EPD can increase the procedural time and the complexity of the procedure (4, 8). Thus, EPD use may be associated with procedure-related complications (9). No-reflow and the periprocedural MI during the SVG interventions have been declining recently. This is mainly because of potent antiplatelet therapy use and improvement in the stents and procedural techniques (10). Due to these conflicting findings, the use of EPD routinely in the SVG intervention has been unclear. As such, there is a need to systematically pool the available evidence and provide clinically meaningful and reliable findings. Accordingly, to clarify the matter, we conducted a systematic review and meta-analysis comparing adverse clinical outcomes such as all-cause mortality, major adverse cardiovascular events (MACE), MI, and target vessel revascularization (TVR) with and without EPD in patients undergoing SVG intervention.

## Methods

### Eligibility Criteria

*Type of studies:* We have included parallel arm individual randomized, quasi-randomized, or cluster RCTs, prospective or retrospective observational studies for the current review. Only published full-text articles or abstracts were eligible for inclusion, while unpublished studies/gray literature were excluded.

*Type of participants:* Studies including patients undergoing PCI in SVG were included.

*Type of intervention:* Studies reporting outcomes separately for the use and non-use of EPD for PCI in SVG were included in our analysis.

*Type of outcome measure:*

- Mortality
- TVR
- Periprocedural MI
- Late MI
- MACE

We have included studies reporting any of the above-mentioned outcomes in both groups.

### Search Strategy

We conducted a comprehensive, systematic, and extensive search in the electronic databases including PubMed Central, Cochrane Library, EMBASE, CINAHL, MEDLINE, Google Scholar, ScienceDirect, and Scopus. Search terms were selected during the protocol stage. Both medical subject headings (MeSH) and free-text words were used to search databases. The terms used were as follows: “Embolic Protection Devices,” “Coronary Artery Disease,” “Percutaneous Coronary Intervention,” “Mortality,” “Target Vessel Revascularization,” “Myocardial Infarction,” “Saphenous Vein Graft,” “Randomized Controlled Trial,” and “Observational Studies.” All terms were used in a variety of combinations and search results were obtained in each individual database. We restricted the search from January 1964 to April 2021 and only those which were published in English.

#### Searching Other Resources

The reference list, of primary studies, was cross-checked to find other studies that satisfy the eligibility criteria of our review process. Additionally, we contacted investigators of the published studies, for clarification or supplementary information required for the quality assessment and outcome measurement of the included studies.

### Data Collection and Analysis

#### Selection of Studies

Three-stage process:

1) Primary screening of title and abstract by two independent investigators by executing the search strategy and retrieving the full-study details.
2) Secondary screening of full study details, of obtained articles, by two independent investigators and filtering based on eligibility criteria of the review.
3) Final selection of studies by a third independent investigator serving as a mediator between the previous two investigators.

#### Data Extraction and Management

Investigators have developed a pre-defined data extraction form, during the protocol stage, which was used by the primary investigator to extract the following information: study title, authors, publication year, study design, participants, settings, the total number of participants in each group, outcome measures, inclusion and exclusion criteria, time of outcome assessment, and details (see next section) necessary for assessing the quality of studies. Extracted data were then transferred into statistical software RevMan version 5.3 (The Cochrane Collaboration and United Kingdom) and the entry was verified for correctness by an independent investigator.

#### Risk of Bias Assessment

The risk of bias was assessed using a Newcastle Ottawa Quality Assessment Form for observational studies under the three domains, namely Selection, Comparability, and Outcome, and Cochrane risk of bias tool for RCTs under the domains generation of random sequence, allocation concealment, blinding of participants, and outcome, incomplete outcome data, and selective reporting of outcome (11, 12). For each of the above-mentioned domains, the risk of bias was graded as low (if adequate information is provided), high (if the information is inadequate or not performed), and unclear (if the information is missing). Studies having a score of 4 or more on the Newcastle Ottawa Scale (or) having low risk of bias in at least four domains in the Cochrane risk of bias tool were considered high quality.

#### Statistical Analysis

Meta-analysis was executed using the software RevMan version 5.3 (Copenhagen: The Nordic Cochrane Center, The Cochrane Collaboration, 2014). Since all the outcomes were dichotomous, a number of events and participants in each group were entered to obtain the pooled effect estimate in terms of odds ratio (OR). We used a random-effects model with inverse-variance (12). In case of missing data, the author of the included trial was contacted and if unable to retrieve the necessary data, an imputation method was implemented.

#### Assessment of Heterogeneity

Evidence of inter-study variance due to heterogeneity was assessed through the chi-square test of heterogeneity and *I*^2^ statistics to quantify the inconsistency. The interpretations for *I*^2^ are as follows: *I*^2^ <25% is mild, 25–75% is moderate and more than 75% is considered as substantial heterogeneity (12). Study-specific and pooled estimates were graphically represented through a forest plot.

#### Assessment of Reporting Biases

Reporting bias was assessed by assessing if the included trial was registered in a trial registry or the full protocol was available. If available, a list of outcomes in the protocol was compared with the list of outcomes mentioned in the full published trial. A funnel plot was performed for the mortality outcome only if other outcomes did not have a requisite number of studies (<10).

#### Additional Analysis

Sensitivity analysis was conducted to assess the robustness of results by removing studies one at a time and checking for significant variation in results. Meta-regression was performed to explore the source of heterogeneity for mortality outcome only as other outcomes did not have a requisite number of studies (<10).

## Results

### Selection of Studies

**Figure 1:**
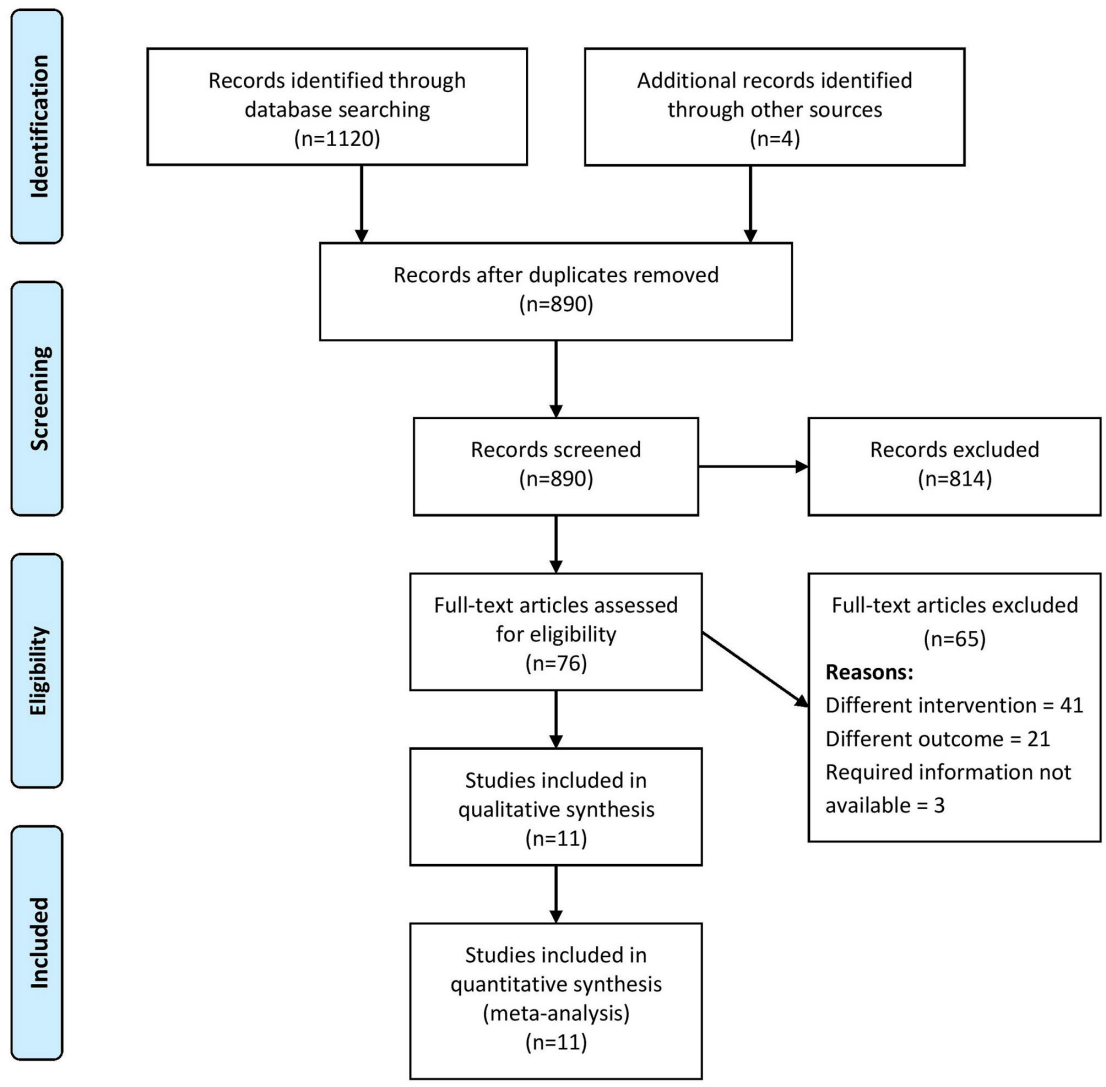
PRISMA flow chart showing the selection of studies for the current review (*n* = 11).

A total of 1,120 records were retrieved through the systematic literature search, of which, 76 studies were relevant for full-text retrieval. An additional four articles were obtained through manual searching of bibliographies within the retrieved studies. During the second screening stage, 11 studies (4–8, 13–18) with a total of 79,009 participants met the eligibility criteria and were included in our review (Figure 1).

### Characteristics of Studies Included

Characteristics of the studies are described in Table 1. In total, 2 out of 11 studies were RCTs, while the remaining studies were observational. The majority of the studies (8/11) were conducted in the USA (6) and Canada (2). In total, 79,009 participants were found in the included studies with sample sizes ranging from 150 to 49,325. The majority of the included studies used Filterwire and Spider as EPD. Follow-up duration ranged from 1 month to 4 years. Among the 11 included studies, all reported on mortality, 9 studies on MACE, 7 studies on late MI and TVR, and 4 studies on periprocedural MI. All the included studies met the high-quality criterion.

**Table 1:** Characteristics of the included studies, *N* = 11.

**No**.	**References**	**Country**	**Study design**	**Sample size in EPD group**	**Sample size in non-EPD group**	**EPD used**	**Mean age (in years)**	**Outcomes assessed**	**Follow-up (in months)**	Quality of the study^*^
1.	Baim et al. (4)	USA	RCT	406	395	Guardwire	EPD = 68 No EPD = 69	Death, MACE, late MI	1	High
2.	Brennan et al. (8)	USA	NCDR CathPCI Registry study	10,432	38,893	SpideRX (*n* = 640), Spider FX (*n* = 558), FilterWire EX (*n* = 3,204) FilterWire EZ (*n* = 6,113) Combination (*n* = 23)	EPD = 75 Non-EPD = 75	Death, MACE, periprocedural MI, late MI, TVR	36	High
3.	Dixon et al. (5)	USA	RCT	173	185	TRAP vascular filter system	EPD = 69.9 No EPD = 70.4	Death, MACE, late MI, TVR	1	High
4.	Golwala et al. (6)	USA	Retrospective	93	71	Proxis (7%), Filterwire (55%), Spider (35%), Guardwire (1%)	EPD = 65.4 No EPD = 67.8	Death, MACE, periprocedural MI, late MI, TVR	12	High
5.	Iqbal et al. (7)	Canada	British columbia cardiac registry	96	1,263	Filterwire	EPD = 74 Non-EPD = 73	Mortality, TVR	24	High
6.	Lavi et al. (13)	Canada	Prospective	198	175	Not provided	EPD = 70.4 No EPD = 69.4	Death, MACE, periprocedural MI, late MI, TVR	36	High
7.	Matar et al. (14)	USA	Retrospective	108	94	Not provided	EPD = 69 No EPD = 68	Death, MACE, late MI, TVR	1	High
8.	Sadr-Ameli et al. (15)	Iran	Prospective	22	128	Not provided	EPD = 63.2 No EPD = 62.5	Death, MACE, periprocedural MI, late MI, TVR	6	High
9.	Shoaib et al. (17)	UK	Retrospective	2,912	17,730	Spider (45%) Filterwire (41%)	EPD = 71 No EPD = 69	Death and MACE	48	High
10.	Valle et al. (18)	USA	Retrospective	2,281	2,401	Filterwire	EPD = 69.2 No EPD = 69.3	Death	1	High
11.	Wańha et al. (16)	Poland	Multicenter registry	190	602	Spider FXTM (50%), FilterWire EZ (29%), EmboShield® (8%), Defender (7%), RX Accunet (3%), Proxis (3%)	EPD = 70 No EPD = 69	Death, MACE, late MI, TVR	12	High

*NA, not available; RCT, randomized controlled trial; USA, United States of America; UK, United Kingdom; EPD, embolic protection devices; MACE, major adverse cardiac event; MI, myocardial infarction*;

* *indicate the use of Newcastle Ottawa scale for quality assessment*.

### Impact of EPD on Adverse Outcomes for PCI Patients on SVG

#### Mortality

**Figure 2:**
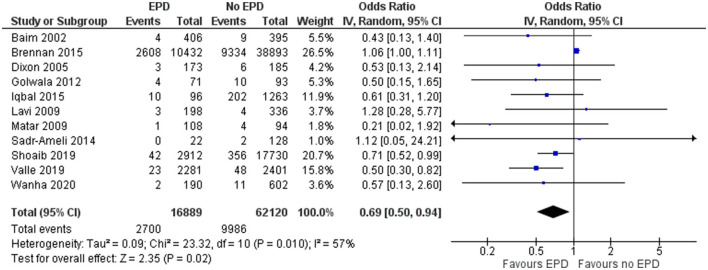
Forest plot showing the difference in mortality between patients with and without embolic protection devices (EPD) following percutaneous coronary intervention (PCI) on saphenous vein grafts (SVG) (*n* = 11).

In total, 11 studies have reported on the mortality outcome among EPD and non-EPD patients. The pooled OR was 0.69 (95% CI: 0.5–0.94) (Figure 2). This indicates that the EPD patients have a significantly lower risk of mortality compared to those patients without EPD (*p* = 0.02). Moderate heterogeneity was calculated between these studies (*I*^2^ = 57%; *p* = 0.01).

Sensitivity analysis was performed by excluding the NCDR CathPCI registry study since this study had the largest sample size compared to the rest of the studies and might skew the final pooled estimate, determined that EPD devices were beneficial with a pooled OR of 0.62 and a CI of 0.49–0.74 and this association was statistically significant (*p* < 0.001). The funnel plot (Supplementary Figure 1) did not show any signs of asymmetry, indicating the absence of publication bias, which was further confirmed by the non-significant Egger's test (*p* = 0.14).

#### Target Vessel Revascularization

**Figure 3:**
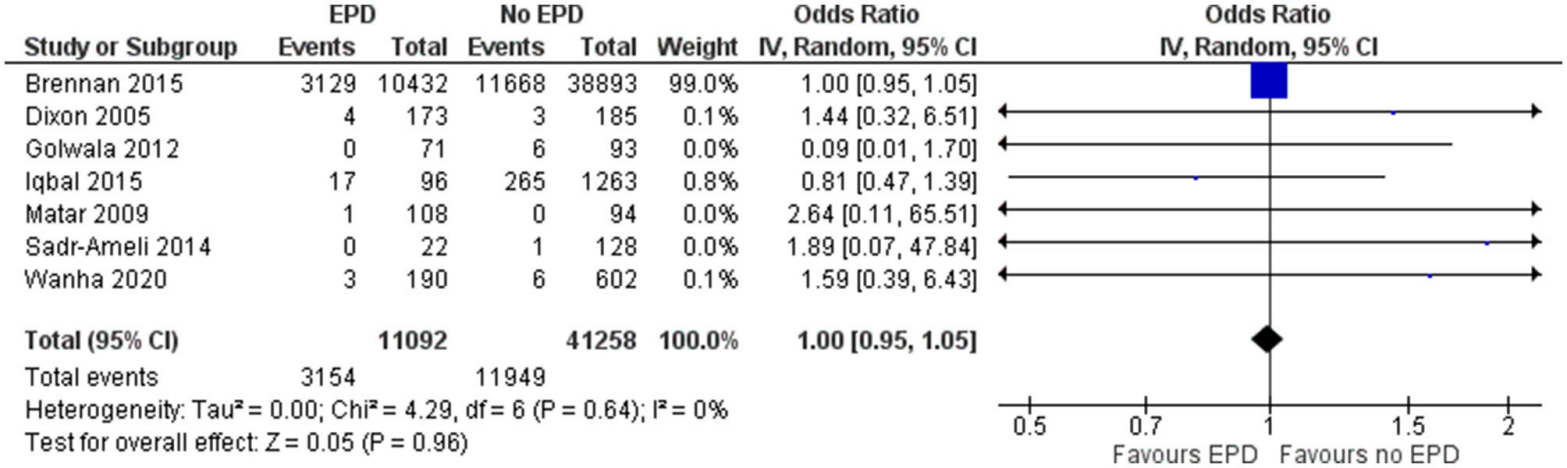
Forest plot showing the difference in target vessel revascularization (TVR) between patients with and without EPD following PCI on SVG (*n* = 7).

In total, 7 studies have reported on the TVR for patients undergoing PCI on SVG in both patients with and without EPD. The pooled OR was 1 (95% CI: 0.95–1.05), indicating no statistically significant difference in terms of TVR for patients with and without EPD following PCI on SVG (Figure 3). There was no heterogeneity among the studies reporting the revascularization outcome (*I*^2^ = 0%, *p* = 0.64). Sensitivity analysis by excluding the NCDR CathPCI registry study revealed no significant change in the pooled estimate in terms of magnitude and direction of association (pooled OR = 0.91; 95% CI: 0.57–1.44).

#### Periprocedural MI

**Figure 4:**
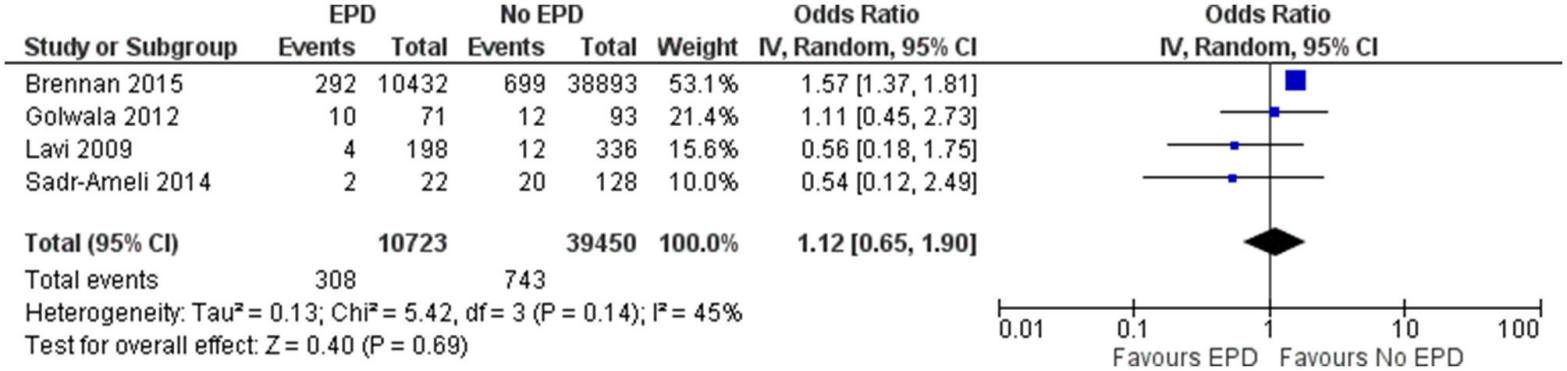
Forest plot showing the difference in periprocedural myocardial infarction (MI) between patients with and without EPD following PCI on SVG (*n* = 4).

In total, 4 studies reported on the periprocedural MI outcome following SVG among patients with and without EPD. The pooled OR was 1.12 (95% CI: 0.65–1.9), indicating that there is no statistically significant difference in periprocedural MI between patients with and without EPD (Figure 4). There was moderate heterogeneity between these studies (*I*^2^ = 45%; *p* = 0.14). Sensitivity analysis by excluding the NCDR CathPCI registry study revealed that there is no significant change in the pooled estimate in terms of magnitude and direction of association (pooled OR = 0.78; 0.41–1.49).

#### Late MI

**Figure 5:**
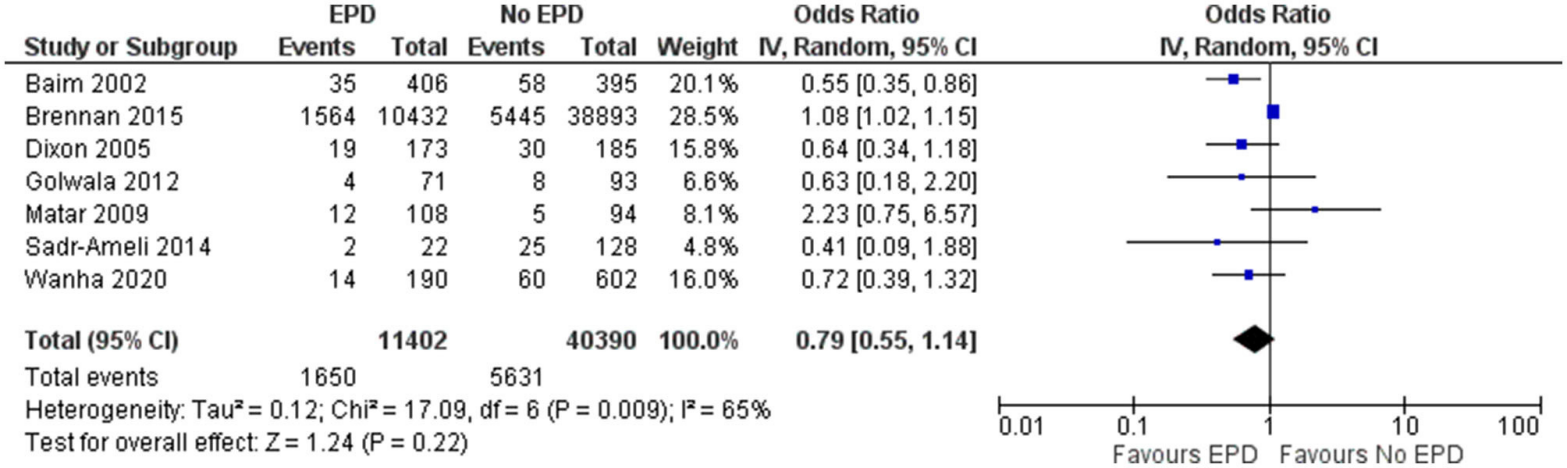
Forest plot showing the difference in late MI between patients with and without EPD following PCI on SVG (*n* = 7).

In total, 7 studies reported on late MI outcomes following SVG among patients with and without EPD. The pooled OR was 0.79 (95% CI: 0.55–1.14), indicating that there is no statistically significant difference in late MI between patients with and without EPD (Figure 5). There was moderate heterogeneity between these studies (*I*^2^ = 65%; *p* = 0.009). Sensitivity analysis was performed by excluding the NCDR CathPCI registry study and found that the EPD devices were beneficial in preventing late MI with pooled OR of 0.68 with a CI of 0.49–0.94 and this association was statistically significant (*p* = 0.02).

#### Major Adverse Cardiac Events

**Figure 6:**
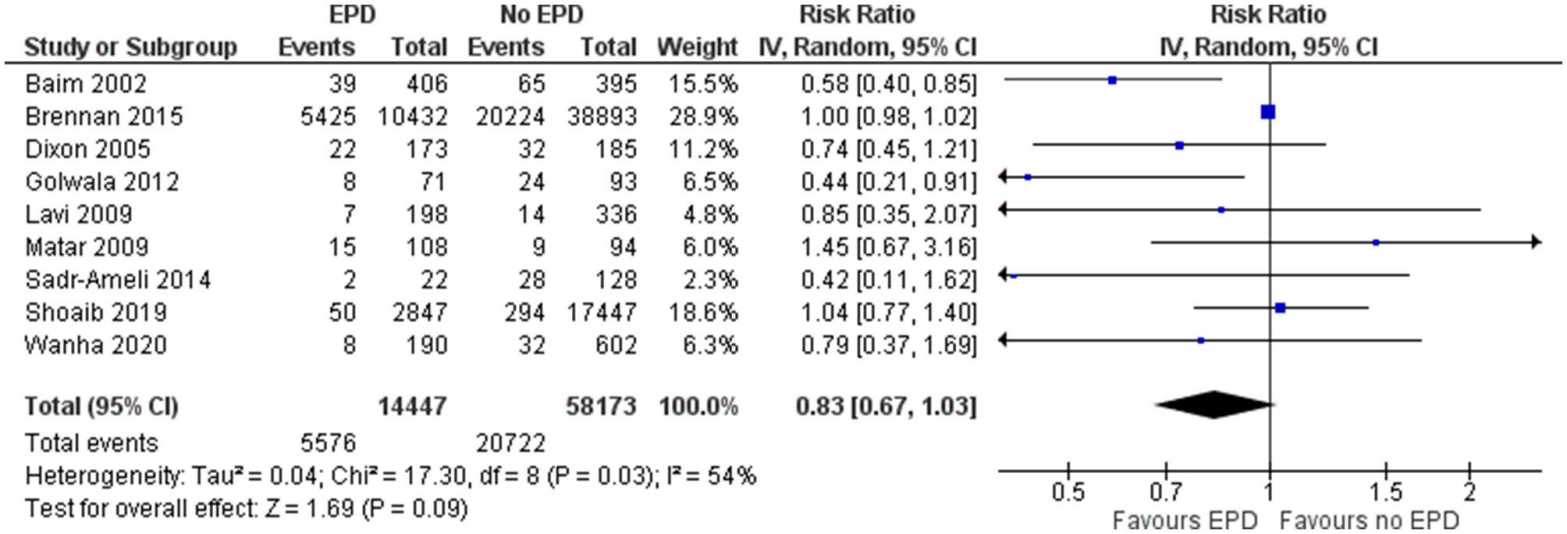
Forest plot showing the difference in major adverse cardiovascular events (MACE) between patients with and without EPD.

In total, 9 studies reported on the MACE outcome following PCI on SVG among patients with and without EPD. The pooled OR was 0.83 (95% CI: 0.67–1.03) (Figure 6), indicating no statistically significant association in MACE between patients with and without EPD. There was significant evidence of heterogeneity among studies reporting the MACE outcome (*I*^2^ = 54%, *p* = 0.03). Sensitivity analysis by excluding the NCDR CathPCI registry study revealed that there is no significant change in the pooled estimate in terms of magnitude and direction of association (pooled OR = 0.77; 95% CI: 0.59–1.01).

## Discussion

Cardiovascular mortality is a major concern for patients undergoing PCI. The risk is higher among patients without EPD when compared to patients with EPD undergoing PCI on SVG (8). However, it is unclear whether EPD can negate this impact and lead to more favorable outcomes. Hence, there is a need for a comprehensive assessment of the impact of EPD on adverse outcomes. This review was conducted with the objective of comparing adverse outcomes in terms of mortality, TVR, periprocedural MI, late MI, and MACE between the patients with and without EPD undergoing PCI on SVG. The most recent data to date was used in conducting this review.

Mortality (pooled OR = 0.69; 95% CI: 0.5–0.94) has been found to be significantly lower among patients with EPD compared to non-EPD patients undergoing PCI on SVG. In addition, sensitivity analysis by excluding the NCDR CathPCI registry study has revealed that the EPD is beneficial in preventing late MI, showing a statistically significant association. Compared with the current study, previous reviews on the impact of EPD on patients undergoing post-PCI SVG revealed similar statistically significant estimates for all the adverse outcomes. There is a variation in the estimates provided by the sensitivity analysis as the current review found EPD beneficial in late MI, while previous reviews found MACE to be beneficial. The possible reason for this discrepancy could be the addition of newer pieces of evidence in our review, skewing the pooled estimate in a different direction. It is surprising to find that EPD is beneficial in reducing mortality, while it is not beneficial to modify the periprocedural MI. EPDs are meant to avoid distal embolization and thereby avoid periprocedural MI. It is mechanistically not possible that the late MIs are reduced by the use of EPD, while the acute periprocedural MI is not prevented. One possible explanation could be that EPD users are generally more thoughtful, take their time in the laboratory to provide quality care without being in a hurry, compliant with all other guidelines (antiplatelet, statins, etc). Still, these findings might be affected by the methodological part of each individual study, sample size, power, and several other statistical factors. It is important to perform more comprehensive trials to find the exact benefit of the EPD following PCI on patients undergoing SVG.

This study identified the benefit of EPD on patients undergoing PCI surgery using SVG. Therefore, it is reasonable to suggest that clinicians should consider the possible use of EPD to avoid adverse complications. The use of this technique has some limitations. Pieces of evidence have suggested that women, post-challenge high blood glucose, insulin resistance, and vulnerability to coronary plaque may cause adverse outcomes following PCI (19–22). EPD utilization has been very limited in the earlier trials. It has evolved with an improved safety profile when using such devices, and it later became a standard of care in routine clinical practice. It should be used with caution as the EPD (especially proximal EPD) suffers from certain limitations such as landing zone restriction, incomplete apposition of the vessel wall, and new aspiration catheters to treat no re-flow (23). To overcome such limitations, several technological advancements have been made such as drug-eluting stents (DES), pharmacology of PCI procedures, advanced procedural techniques, intravascular imaging, etc (24, 25). These techniques and procedures might finally mitigate the benefits of EPD on the saphenous venous graft PCI results. Till comprehensive pieces of evidence are generated on these newer advancements, they cannot fully replace EPD in routine clinical practice.

Our results should be interpreted with caution and inferred accordingly, considering the difference in methods and quality across included studies. In our analysis, we found significant inter-study variability (significant chi-square test for heterogeneity and moderate to high *I*^2^ statistics) for most outcomes except TVR and peri-procedural MI. The reason for such high heterogeneity in outcomes can be attributed to the methodological differences between the included studies.

This study increases the knowledge on the impact of EPD on the adverse outcomes post-PCI on patients undergoing SVG. We are aware of the limitations in our review. We included both RCTs and observational studies in our review. The inclusion of these mixed study designs makes it difficult to infer the causal associations between the exposure and outcomes. More RCTs with larger sample sizes are necessary to gather more credible evidence. We could not perform a funnel plot to assess the publication bias for all the outcomes except mortality due to the limitation in the number of studies. Finally, most of the studies included in our review were conducted in high-income countries, which may limit the generalizability of our findings to other geographical regions.

Despite all these limitations, our study has several important implications for clinicians during routine practice. The use of EPD may have a positive impact on cardiovascular outcomes, especially amongst patients undergoing post-PCI SVG. Previously, there was inconsistency around EPD impact given mixed reports by previous studies. Our study clarifies this inconsistency and provided a reliable pooled estimate. More longitudinal research studies are required for accurately assessing the impact of EPD following PCI on patients undergoing SVG. Future studies should focus on how these interventions will perform with pre-medications like Nipride, Nicardipine, or dual antiplatelet therapy, as early administration of these drugs has been shown to improve the clinical outcomes of the patients undergoing PCI (26). Nonetheless, it is reasonable to utilize the EPDs in cases where device placement can be considered safe and feasible unless a new trial demonstrates convincing evidence saying that the EPDs are not at all beneficial.

## Data Availability Statement

The raw data supporting the conclusions of this article will be made available by the authors, without undue reservation.

## Author Contributions

JY, JZ, JN, and WS conceived and designed the study. JY, JZ, and YF were involved in literature search, data collection, and wrote the paper. WS and SF analyzed the data, reviewed, and edited the manuscript. All authors have read and approved the final manuscript.

## Conflict of Interest

The authors declare that the research was conducted in the absence of any commercial or financial relationships that could be construed as a potential conflict of interest.

## Publisher's Note

All claims expressed in this article are solely those of the authors and do not necessarily represent those of their affiliated organizations, or those of the publisher, the editors and the reviewers. Any product that may be evaluated in this article, or claim that may be made by its manufacturer, is not guaranteed or endorsed by the publisher.

## Supplementary Material

Supplementary Figure 1 Funnel plot for checking publication bias for mortality outcome.

## References

[1] Hong MK Mehran R Dangas G Mintz GS Lansky AJ Pichard AD . Creatine kinase-MB enzyme elevation following successful saphenous vein graft intervention is associated with late mortality. Circulation. (1999) 100:2400–5. 10.1161/01.CIR.100.24.2400 10595951

[2] Piana RN Paik GY Moscucci M Cohen DJ Gibson CM Kugelmass AD . Incidence and treatment of “no-reflow” after percutaneous coronary intervention. Circulation. (1994) 89:2514–8. 10.1161/01.CIR.89.6.2514 8205658

[3] Levine GN Bates ER Blankenship JC Bailey SR Bittl JA Cercek B . 2011 ACCF/AHA/SCAI Guideline for Percutaneous Coronary Intervention: a report of the American College of Cardiology Foundation/American Heart Association Task Force on Practice Guidelines and the Society for Cardiovascular Angiography and Interventions. Circulation. (2011) 124:e574–651. 10.1002/ccd.23390 22064601

[4] Baim DS Wahr D George B Leon MB Greenberg J Cutlip DE . Randomized trial of a distal embolic protection device during percutaneous intervention of saphenous vein aorto-coronary bypass grafts. Circulation. (2002) 105:1285–90. 10.1161/01.CIR.0000012783.63093.0C 11901037

[5] Dixon SR Mann JT Lauer MA Casale PN Dippel EJ Strumpf RK . A randomized, controlled trial of saphenous vein graft intervention with a filter-based distal embolic protection device: TRAP trial. J Interv Cardiol. (2005) 18:233–41. 10.1111/j.1540-8183.2005.00039.x 16115151

[6] Golwala H Hawkins BM Stavrakis S Abu-Fadel MS. Embolic protection device use and outcomes in patients receiving saphenous vein graft interventions–a single-center experience. J Invasive Cardiol. (2012) 24:1–3. 22210580

[7] Iqbal MB Nadra IJ Ding L Fung A Aymong E Chan AW . Embolic protection device use and its association with procedural safety and long-term outcomes following saphenous vein graft intervention: an analysis from the British Columbia Cardiac registry. Catheter Cardiovasc Interv. (2016) 88:73–83. 10.1002/ccd.26237 26482020

[8] Brennan JM Al-Hejily W Dai D Shaw RE Trilesskaya M Rao SV . Three-year outcomes associated with embolic protection in saphenous vein graft intervention: results in 49 325 senior patients in the Medicare-linked National Cardiovascular Data Registry CathPCI Registry. Circ Cardiovasc Interv. (2015) 8:e001403. 10.1161/CIRCINTERVENTIONS.114.001403 25714391

[9] Abdel-Karim A-RR Papayannis AC Mahmood A Michael TT Rangan BV Makke L . Role of embolic protection devices in ostial saphenous vein graft lesions. Catheter Cardiovasc Interv. (2012) 80:1120–6. 10.1002/ccd.23471 22422709

[10] Lee MS Park S-J Kandzari DE Kirtane AJ Fearon WF Brilakis ES . Saphenous vein graft intervention. JACC Cardiovasc Interv. (2011) 4:831–43. 10.1016/j.jcin.2011.05.014 21851895

[11] Wells G Shea B O'Connell D Peterson je Welch V Losos M. The Newcastle–Ottawa Scale (NOS) for Assessing the Quality of Non-Randomized Studies in Meta-Analysis? Ottawa (2000). Available Online at: http://www.ohri.ca/programs/clinical_epidemiology/oxford.asp

[12] Higgins J Green S. Cochrane Handbook for Systematic Reviews of Interventions. United Kingdom: The Cochrane Collaboration and John Wiley & Sons (2009). 10.1002/9780470712184

[13] Lavi S Ivanov J Appleby CE Seidelin PH Mackie K Schwartz L . Selective use of embolic protection devices during saphenous vein grafts interventions: a single-center experience. Catheter Cardiovasc Interv. (2010) 75:1037–44. 10.1002/ccd.22392 20517966

[14] Matar FA Smith K Rossi P Vandormael M Sullebarger JT Taylor M . Limitations of embolic protection in saphenous vein graft intervention: insights from 202 consecutive patients. J Interv Cardiol. (2009) 22:240–6. 10.1111/j.1540-8183.2009.00465.x 19490353

[15] Sadr-Ameli M Mousavi H Heidarali M Maadani M Ghelich Y Ghadrdoost B. Early and midterm major adverse cardiac events in patient with saphenous vein graft using direct stenting or embolic protection device stenting. Res Cardiovasc Med. (2014) 3:e13012. 10.5812/cardiovascmed.13012 25478526 PMC4253743

[16] Wańha W Mielczarek M Gilis-Malinowska N Roleder T Milewski M Ładziński S . Safety and efficacy of embolic protection devices in saphenous vein graft interventions: a propensity score analysis-multicenter SVG PCI PROTECTA study. J Clin Med. (2020) 9:1198. 10.3390/jcm9041198 32331299 PMC7230434

[17] Shoaib A Kinnaird T Curzen N Ludman P Smith D Khoo CW . Outcomes following percutaneous coronary intervention in saphenous vein grafts with and without embolic protection devices. JACC Cardiovasc Interv. (2019) 12:2286–95. 10.1016/j.jcin.2019.08.037 31753300

[18] Valle JA Glorioso TJ Schuetze KB Grunwald GK Armstrong EJ Waldo SW. contemporary use of embolic protection devices during saphenous vein graft intervention. Circ Cardiovasc Interv. (2019) 12:e007636. 10.1161/CIRCINTERVENTIONS.118.007636 31014092

[19] Trifunovic D Stankovic S Sobic-Saranovic D Marinkovic J Petrovic M Orlic D . Acute insulin resistance in ST-segment elevation myocardial infarction in non-diabetic patients is associated with incomplete myocardial reperfusion and impaired coronary microcirculatory function. Cardiovasc Diabetol. (2014) 13:73. 10.1186/1475-2840-13-73 24708817 PMC4234386

[20] Iguchi T Hasegawa T Otsuka K Matsumoto K Yamazaki T Nishimura S . Insulin resistance is associated with coronary plaque vulnerability: insight from optical coherence tomography analysis. Eur Heart J Cardiovasc Imaging. (2014) 15:284–91. 10.1093/ehjci/jet158 24022065

[21] Lopez-de-Andres A Jimenez-García R Hernandez-Barrera V Perez-Farinos N de Miguel-Yanes JM Mendez-Bailon M . National trends in utilization and outcomes of coronary revascularization procedures among people with and without type 2 diabetes in Spain (2001-2011). Cardiovasc Diabetol. (2014) 13:3. 10.1186/1475-2840-13-3 24383412 PMC3881504

[22] Kuramitsu S Yokoi H Domei T Nomura A Watanabe H Yamaji K . Impact of post-challenge hyperglycemia on clinical outcomes in Japanese patients with stable angina undergoing percutaneous coronary intervention. Cardiovasc Diabetol. (2013) 12:74. 10.1186/1475-2840-12-74 23651930 PMC3651729

[23] Azfar G Zaman Jagath Herath. Chapter 25 - percutaneous coronary intervention in saphenous vein graft disease. In: Norell MS Perrins J Meier B Lincoff AM, editors. Essential Interventional Cardiology. 2nd ed. W.B. Saunders: Philadelphia (2008). p. 309–18. 10.1016/B978-0-7020-2981-3.50028-3

[24] Hong YJ Pichard AD Mintz GS Kim SW Lee SY Kim SY . Outcome of undersized drug-eluting stents for percutaneous coronary intervention of saphenous vein graft lesions. Am J Cardiol. (2010) 105:179–85. 10.1016/j.amjcard.2009.09.006 20102915

[25] Costa F Cohen DJ. Embolic protection devices in saphenous vein graft intervention. Circ Cardiovasc Interv. (2019) 12:e007879. 10.1161/CIRCINTERVENTIONS.119.007879 31014091

[26] Kumar V Sharma AK Kumar T Nath RK. Large intracoronary thrombus and its management during primary PCI. Indian Heart J. (2020) 72:508–16. 10.1016/j.ihj.2020.11.009 33357638 PMC7772595

